# Clinical, Microbiological, and Hematological Characteristics of Pediatric Brucellosis in Saudi Arabia: A Single-Center Retrospective Study

**DOI:** 10.3390/diagnostics16121807

**Published:** 2026-06-11

**Authors:** Nawaf R. R. Alshammari, Fahaad S. Alenazi, Mohd Saleem, Nahed Fathallah Fahmy Mohamed, Saada A. Alogla, Najd B. Albalawi, Noor Munawer Alrashidi, Layan Zaid Alhamashi, Abdulelah Ghazi AlHarbi, Khalid Ata Alshammari, Misheal Ayed Alshammeri, Azharuddin Sajid Syed Khaja

**Affiliations:** 1 Department of Pediatrics, College of Medicine, University of Ha’il, Ha’il 81422, Saudi Arabia; nar.alshammari@uoh.edu.sa; 2 Department of Pharmacology, College of Medicine, University of Ha’il, Ha’il 81422, Saudi Arabia; fs.alenazi@uoh.edu.sa; 3 Medical Education Committee, College of Medicine, University of Ha’il, Ha’il 81422, Saudi Arabia; 4 Department of Pathology, College of Medicine, University of Ha’il, Ha’il 81422, Saudi Arabia; m.saleem@uoh.edu.sa; 5 Department of Microbiology, Maternity and Children Hospital, Ha’il 81422, Saudi Arabia; dr.nahedfahmy@gmail.com; 6 Department of Microbiology & Immunology, Faculty of Medicine, Sohag University, Sohag 82524, Egypt; 7 College of Medicine, University of Ha’il, Ha’il 81422, Saudi Arabia; s202001604@uoh.edu.sa (S.A.A.); s202007050@uoh.edu.sa (N.B.A.); s202100026@uoh.edu.sa (N.M.A.); s202100193@uoh.edu.sa (L.Z.A.); s202008132@uoh.edu.sa (A.G.A.); s201902974@uoh.edu.sa (K.A.A.); s202003716@uoh.edu.sa (M.A.A.)

**Keywords:** brucellosis, hematological manifestations, thrombocytopenia, pancytopenia

## Abstract

**Background and Objectives:** Brucellosis remains a significant zoonotic disease in endemic regions such as Saudi Arabia, with children being particularly vulnerable. Pediatric brucellosis often presents with nonspecific symptoms, and hematological abnormalities can serve as important yet underrecognized diagnostic clues. This study aimed to evaluate the demographic, clinical, microbiological, treatment, and hematological characteristics of patients with pediatric brucellosis in the Hail region of Saudi Arabia and to assess the diagnostic value of hematological parameters. **Methods:** This retrospective observational study included children aged ≤15 years who were diagnosed with brucellosis at a tertiary care hospital in Hail between 2014 and 2025. Demographic, clinical, laboratory, microbiological, and treatment data were analyzed. Hematological parameters were compared between culture-confirmed and non-culture-confirmed brucellosis cases using multivariate and receiver operating characteristic (ROC) analyses. **Results:** A total of 38 pediatric patients were included (mean age 8.6 years; 57.9% male). Positive culture results were observed in 42.1% of the cases, with *Brucella melitensis* being the predominant species (68.75%). Fever (89.5%) and bone/joint pain (71.1%) were the most frequent symptoms. Culture-confirmed brucellosis patients had significantly lower hemoglobin levels (10.8 vs. 12.1 g/dL; *p* = 0.020), white blood cell counts (*p* = 0.046), and absolute neutrophil counts (*p* = 0.037). ROC analysis revealed a fair diagnostic performance for hemoglobin (AUROC = 0.695), WBC (0.699), and ANC (0.680). Leukopenia demonstrated high specificity (95.5%) and positive predictive value. **Conclusions:** Pediatric brucellosis is commonly associated with anemia, leukopenia, and neutropenia. Although no single hematological parameter independently predicts infection, the combination of these abnormalities may support early clinical suspicion, particularly in resource-limited endemic settings.

## 1. Introduction

Brucellosis is among the most common zoonotic infections worldwide and continues to pose significant public health challenges, particularly in endemic areas such as the Middle East, the Mediterranean Basin, and parts of Asia [1,2]. Despite advances in public health surveillance and control measures, the disease persists due to the establishment of animal reservoirs, ongoing consumption of unpasteurized dairy products, and sustained human–animal contact in rural communities. Within this epidemiological landscape, children represent a particularly vulnerable population, as exposure frequently occurs within household settings, and their clinical presentations are often nonspecific, contributing to diagnostic delays and under-recognition of the disease [1].

Although pediatric brucellosis has been characterized as an “almost forgotten disease”, it remains clinically relevant in endemic countries where transmission continues [2]. Affected children usually present with symptoms such as prolonged or intermittent fever, malaise and weight loss, arthralgia, hepatosplenomegaly, and lymphadenopathy. However, these symptoms often overlap significantly with those of many other infectious, inflammatory, and blood disorders, making diagnosis challenging for clinicians [3,4]. This difficulty is especially noticeable when there is no clear history of exposure to risk factors, highlighting the crucial role of laboratory findings, particularly hematological abnormalities, in increasing clinical suspicion.

Hematological manifestations are among the most important yet frequently underappreciated aspects of pediatric brucellosis. Accumulating evidence indicates that *Brucella* infection can affect one or more hematopoietic cell lines, resulting in anemia, leukopenia, leukocytosis, thrombocytopenia, or pancytopenia [5,6]. These abnormalities may range from mild and transient to severe presentations that mimic primary hematological disorders, including leukemia and bone marrow failure syndromes. The concept of “hematological footprints” has thus emerged to describe the diverse spectrum of blood-related changes associated with *Brucella* infection in the pediatric population.

Large pediatric cohort studies from endemic regions have substantially advanced our understanding of these hematological patterns. A landmark study involving 622 children from Eastern Turkey revealed anemia as the most frequent hematological finding, followed by thrombocytopenia and leukopenia, with pancytopenia observed in a notable proportion of patients [7]. Subsequent pediatric series have corroborated these findings, reinforcing the notion that hematological involvement is not merely an occasional complication but also an integral component of the disease spectrum [8,9]. The underlying pathophysiological mechanisms are multifactorial, encompassing bone marrow suppression, hypersplenism, immune-mediated peripheral destruction, and in rare instances, hemophagocytic activity [9].

In Saudi Arabia, brucellosis remains endemic despite longstanding national control programs. Epidemiological surveillance data reveal sustained reporting of human brucellosis cases over the past two decades, with notable regional variation in disease burden [10,11]. Multiple studies have indicated that children constitute a substantial proportion of affected individuals, who frequently present with atypical clinical features or complicated disease courses [4,12]. Pediatric case series from various regions of Saudi Arabia consistently identify hematological abnormalities as common laboratory findings, although these abnormalities are typically described as secondary observations rather than being systematically evaluated as potential diagnostic indicators [3,13].

The diagnosis of brucellosis depends on clinical suspicion supported by serological testing and blood culture confirmation. However, conventional serological methods, including the standard agglutination test, present well-recognized limitations, particularly during early infection and in bacteremic patients [14]. While advances in laboratory diagnostics have improved both sensitivity and specificity, diagnostic delays remain common when children present predominantly with hematological abnormalities [15,16]. The misinterpretation of cytopenias in this context may precipitate unnecessary and extensive investigations, delay appropriate antimicrobial therapy, and contribute to increased morbidity.

Recognition of the hematological profile characteristics of pediatric brucellosis, therefore, has substantial clinical importance. Awareness of features such as anemia with thrombocytopenia or unexplained pancytopenia in febrile children from endemic areas can prompt earlier consideration of brucellosis in the differential diagnosis [6,9]. Early diagnosis followed by appropriate antimicrobial treatment has been associated with rapid normalization of hematological parameters and favorable outcomes [17].

Although numerous international studies have characterized the hematological manifestations of pediatric brucellosis, single-center experiences continue to provide valuable insights. Such studies reflect local epidemiological patterns, diagnostic practices, and disease behavior, which may vary considerably across different geographic regions [18,19]. In endemic areas, detailed assessments of hematological findings can increase clinician awareness and improve diagnostic accuracy, ultimately facilitating timely intervention and reducing disease-related morbidity.

Several studies from different regions of Saudi Arabia, including Najran [12], Riyadh [4,20], and the Eastern Province [21], have described the epidemiological and clinical characteristics of pediatric brucellosis. However, these investigations have primarily focused on clinical presentation, risk factors, and treatment outcomes, while the diagnostic significance of hematological abnormalities has received comparatively limited attention. Regional differences in livestock exposure patterns, healthcare-seeking behavior, and disease burden may also influence the clinical and laboratory manifestations of the disease. Moreover, data on the hematological profile of pediatric brucellosis remains scarce within the Hail region. To address this knowledge gap, the present study was conducted at a tertiary care hospital in the Hail region of Saudi Arabia. Our main goals were to determine the prevalence, clinical features, microbiological profile, and treatment approaches for pediatric brucellosis in this population. Additionally, we aimed to thoroughly examine the range of hematological signs in these children and to evaluate the diagnostic value of certain hematological parameters, such as hemoglobin levels, using receiver operating characteristic (ROC) and regression analyses.

## 2. Materials and Methods

### 2.1. Study Design and Setting

A retrospective observational study was conducted at the Maternity and Children’s Hospital in Hail, a major tertiary care and referral center that provides specialized pediatric services to patients from Hail city and the surrounding geographical areas. The retrospective design facilitated the analysis of confirmed pediatric brucellosis cases by utilizing existing clinical and laboratory records accumulated over an extended period.

### 2.2. Study Population

The study population included all children aged 15 years and younger who were diagnosed with brucellosis at the Maternity and Children’s Hospital during the study period.

### 2.3. Inclusion and Exclusion Criteria

Children aged ≤15 years with a diagnosis of brucellosis based on compatible clinical findings together with either culture-confirmed infection (isolation of *Brucella* spp. from blood culture) or serological evidence (Standard Tube Agglutination Test [STAT] titer ≥ 1:160), and for whom complete medical records were available, were included in the study. These diagnostic criteria were applied in accordance with the Centers for Disease Control and Prevention (CDC) case definitions for brucellosis. Patients were excluded if their medical records were incomplete or if their brucellosis diagnosis was established outside the specified study timeframe.

### 2.4. Study Period and Data Collection

Medical records from the Pediatric Department and the Department of Microbiology were systematically reviewed for the period spanning from 2014 to 2025. Data extracted from patient records included the participants’ demographic characteristics, clinical presentation (symptoms, signs, and illness duration), laboratory findings, microbiological results (culture and serology), treatment details, and clinical outcomes.

### 2.5. Laboratory Diagnosis of Brucellosis

Laboratory diagnosis of brucellosis was based on isolation of the organism from blood culture and serological testing for antibodies. For culture, the commercial blood culture system BD BACTEC^TM^ (Becton Dickinson, Franklin Lakes, NJ, USA) was used. According to the manufacturer’s instructions, blood samples (1–3 mL) were collected aseptically and inoculated into BD BACTEC^TM^ Peds Plus/F bottles. BD BACTEC^TM^ has a continuous monitoring system for bacterial growth that can detect the organism within 5–7 days, with bottles incubated for up to 14 days. Positive blood culture bottles were subsequently examined by direct Gram stain and subcultured on blood agar and chocolate agar. All specimen and culture handling were performed in Biological Safety Cabinet 2. Inoculated plates were incubated at 37 °C in 5–10% CO_2_, and in a humid atmosphere. As a preliminary identification of *Brucella* spp. by direct Gram smear, *Brucella* appeared as small Gram-negative coccobacilli. By culture, colonies appeared small, convex, gamma-hemolytic, translucent after 48 h of incubation. By biochemical reactions, *Brucella* spp. was catalase-positive, urease-positive, and oxidase-positive. Identification was confirmed using the VITEK 2 compact system (BioMérieux, Marcy-L’Étoile, France).

Serological diagnosis of Brucellosis was performed by the Standard Tube Agglutination Test (STAT). Three to five milliliters of blood were collected by venipuncture and transferred into a sterile, anticoagulant-free tube. The tubes were centrifuged at 3000 rpm for 5 min, and the serum was used for testing. Serial dilution of patient serum was performed by adding 1.9 mL of 0.85% NaCl solution to tube 1 and 1.0 mL to tubes 2–10. Then, 100 μL of serum was added to tube 1 and mixed well. Twofold serial dilutions were prepared by transferring 1.0 mL from one tube to the next, mixing well after each transfer; 1.0 mL from the last tube was discarded. The dilutions of the tubes were 1:20, 1:40, up to 1:10,240, respectively. Then, 50 μL of *Brucella* meltiness and *Brucella abortus* antigens were added separately to all tubes, including the control. The racks were incubated in a water bath at 37 °C for 24 h.

The tubes were observed for agglutination. The titer was measured as the highest dilution in the tube that showed positive agglutination. In accordance with the established diagnostic criteria, antibody titers ≥1:160 in the presence of compatible clinical manifestations were considered diagnostic for active brucellosis. If the initial tests were negative in patients with a clinical picture of brucellosis, STAT was repeated after 4 weeks. Polymerase chain reaction (PCR) testing was not routinely performed during the study period.

### 2.6. Assessment of Hematological Parameters

Hematological data, including hemoglobin levels, total and differential white blood cell counts, and platelet counts, were obtained from complete blood count reports. Anemia was defined according to age-specific World Health Organization (WHO) hemoglobin cutoff criteria. Leukopenia and thrombocytopenia were defined according to age-specific reference ranges used by the hospital laboratory. Pancytopenia was defined as the simultaneous presence of anemia, leukopenia, and thrombocytopenia.

### 2.7. Statistical Analysis

The data were analyzed using SPSS software version 25 (IBM Corporation, Armonk, NY, USA). Descriptive statistics, including frequencies, percentages, means, and standard deviations, were used to summarize the demographic, clinical, microbiological, and hematological characteristics. Independent-samples *t*-tests were used to compare hematological parameters between culture-confirmed brucellosis and non-culture-confirmed brucellosis patients. One-way analysis of variance (ANOVA) was used to compare hematological parameters among different *Brucella* species groups. Chi-square tests were used to assess associations between categorical variables, including clinical symptoms and Brucella infection status. Logistic regression analysis was performed to evaluate the independent predictive value of hematological parameters for culture-confirmed brucellosis, while ROC curve analysis was used to assess their diagnostic performance and determine optimal cutoff values. A *p*-value < 0.05 was considered statistically significant.

## 3. Results

### 3.1. Demographic Characteristics of the Study Participants

A total of 38 pediatric patients with brucellosis were included in the study. The mean age of the participants was 8.6 years (range: 3–15 years). The distribution across age groups was as follows: 3–6 years (*n* = 10, 26.3%), >6–9 years (*n* = 11, 28.9%), >9–12 years (*n* = 10, 26.3%), and >12–15 years (*n* = 7, 18.4%) (Table 1). The majority of the patients were male (57.9%, *n* = 22), with a male-to-female ratio of approximately 1.4:1. All participants were Saudi nationals.

### 3.2. Clinical Presentation of the Study Participants

**Figure 1:**
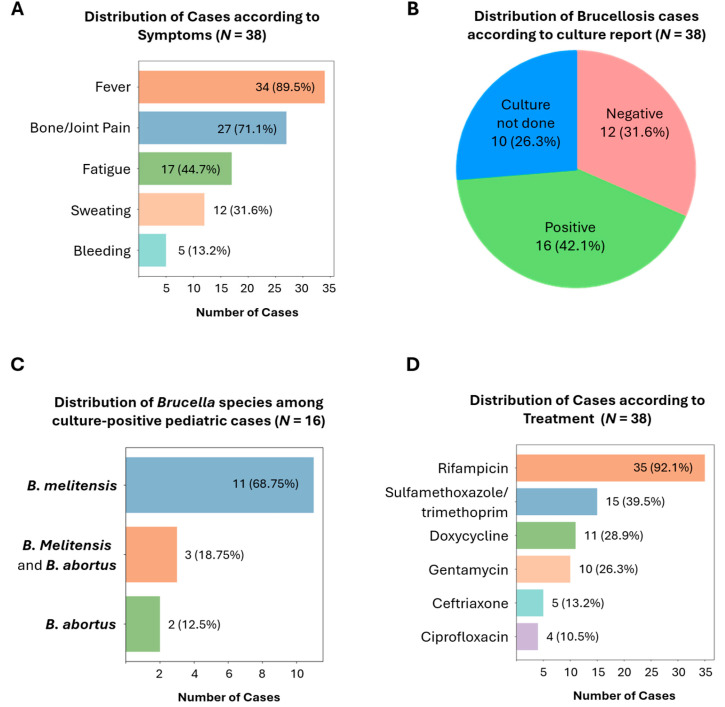
Clinical, microbiological, and treatment characteristics of pediatric brucellosis patients (*n* = 38), showing the distribution of presenting symptoms (**A**), culture report outcomes (**B**), *Brucella* species among culture-positive cases (**C**), and treatment patterns in affected patients (**D**). Data are presented as the number and percentage of patients for each category.

Fever was the most common presenting symptom, observed in 89.5% (*n* = 34) of patients (Figure 1A). Musculoskeletal involvement, including bone and joint pain, was highly prevalent, affecting 71.1% (*n* = 27) of participants. Constitutional symptoms were also frequent, with fatigue reported in 44.7% (*n* = 17) and sweating in 31.6% (*n* = 12) of the cases. Bleeding manifestations were uncommon, observed in only 13.2% (*n* = 5) of patients.

### 3.3. Microbiological Profile

#### 3.3.1. Blood Culture Results

Blood cultures were performed in 28 (73.7%) patients (Figure 1B). Among those tested, positive cultures were obtained in 16 cases (42.1%), while 12 cases (31.6%) yielded negative results. Culture testing could not be performed for 10 (26.3%) participants. Accordingly, subsequent analyses compared patients with culture-confirmed brucellosis (*n* = 16) and non-culture-confirmed brucellosis (*n* = 22).

#### 3.3.2. Brucella Species Identification

Among the 16 culture-positive cases, *Brucella melitensis* was the predominant species, accounting for 11 (68.75%) isolates (Figure 1C). *B. abortus* alone was detected in 2 (12.5%) cases, whereas mixed infection with both *B. melitensis* and *B. abortus* was detected in 3 (18.75%) cases.

#### 3.3.3. Antimicrobial Treatment Patterns

Combination antimicrobial therapy was employed in all patients, with rifampicin being the most frequently prescribed agent (92.1%, *n* = 35) (Figure 1D). Sulfamethoxazole/trimethoprim was used in 39.5% (*n* = 15) of the patients, followed by doxycycline in 28.9% (*n* = 11) and gentamicin in 26.3% (*n* = 10). Ceftriaxone and ciprofloxacin were used less frequently, in 13.2% (*n* = 5) and 10.5% (*n* = 4) of the cases, respectively.

### 3.4. Hematological Profiles and Comparisons Between Culture-Confirmed Brucellosis and Non-Culture-Confirmed Brucellosis Patients

The hematological profile revealed significant differences in hemoglobin (Hb) levels, white blood cell (WBC) counts, and absolute neutrophil counts (ANCs) between non-culture-confirmed brucellosis and culture-confirmed brucellosis individuals (Table 2). The mean hemoglobin concentration was significantly lower in the culture-confirmed brucellosis group (10.8 ± 1.7 g/dL) than in the non-culture-confirmed brucellosis group (12.1 ± 1.4 g/dL), with the difference reaching statistical significance (*p* = 0.020), suggesting an association between brucellosis and anemia.

The total WBC count was also significantly lower in the culture-confirmed brucellosis patients (5.0 ± 2.0 × 10^3^/µL) than in the non-culture-confirmed brucellosis patients (6.2 ± 1.7 × 10^3^/µL) (*p* = 0.046). Similarly, the ANC was significantly lower in the culture-confirmed brucellosis patients (1475.8 ± 745.0 cells/µL) than in the non-culture-confirmed brucellosis patients (2025.9 ± 789.2 cells/µL) (*p* = 0.037), indicating relative neutropenia in the infected group.

However, no statistically significant differences in absolute monocyte count (AMC), absolute lymphocyte count (ALC), or platelet count (PLT) were detected between the two groups (*p* > 0.05 for all). Although the AMC and PLT values were lower in culture-confirmed brucellosis individuals and the ALC values were slightly higher, these differences did not reach statistical significance.

### 3.5. Comparison of Hematological Parameters Across Brucella Species

Comparisons of hematological indices among patients infected with *B. melitensis*, *B. abortus*, and mixed *B. melitensis* and *B. abortus* strains revealed no statistically significant differences across all evaluated parameters (*p* > 0.05) (Table 3). Hemoglobin levels varied slightly among the groups, with mean values of 10.9 ± 1.7 g/dL in *B. melitensis*, 11.8 ± 0.6 g/dL in *B. abortus*, and 9.4 ± 2.3 g/dL in mixed infections; however, these differences were not significant (F = 1.05, *p* = 0.377). Similarly, WBC counts, ANC, AMC, and ALC varied widely but did not differ significantly among species groups (*p* > 0.05 for all). Platelet counts also did not differ significantly, despite higher mean values in *B. abortus* cases than in the other groups.

### 3.6. Associations Between Clinical Symptoms and Brucella Infection

The association between common presenting symptoms and Brucella infection status was assessed using chi-square analysis (Table 4). Fever was frequently reported in both the non-culture-confirmed brucellosis (55.9%) and culture-confirmed brucellosis (44.1%) patients, with no significant association observed (*p* = 0.464; OR = 0.42; 95% CI: 0.04–4.48). Fatigue was also not significantly associated with the culture-confirmed brucellosis, which was present in 35.3% of the culture-confirmed brucellosis patients (*p* = 0.444; OR = 1.67; 95% CI: 0.45–6.19).

Bone and joint pain differed significantly between the two groups and were more frequently reported among patients with non-culture-confirmed brucellosis than among those with culture-confirmed brucellosis. Bone/joint pain was present in 20 out of 27 symptomatic patients in the non-culture-confirmed group compared with 7 out of 27 symptomatic patients in the culture-confirmed group (χ^2^ = 10.02, *p* = 0.002; OR = 12.86, 95% CI: 2.22–74.54). This OR reflects the increased odds of bone/joint pain in the non-culture-confirmed group. Therefore, this finding should be interpreted as a greater frequency of musculoskeletal symptoms among non-culture-confirmed cases rather than as a protective or negative association.

Sweating did not differ significantly between groups (χ^2^ = 2.11; *p* = 0.147; OR = 3.00; 95% CI: 0.66–13.66), although a numerically greater percentage of non-culture-confirmed brucellosis patients reported it (75.0% vs. 25.0%). Bleeding also showed no statistically significant association (χ^2^ = 3.39, *p* = 0.066; OR = 0.14, 95% CI: 0.01–1.43), although the trend suggested a potential association with culture-confirmed brucellosis that did not reach statistical significance.

### 3.7. Multivariate Analysis of Hematological Parameters

Multivariate analysis of variance (MANOVA) was performed to assess the overall effect of Brucella infection status on hematological parameters (Table 5). None of the individual hematological parameters demonstrated a statistically significant effect at the 5% significance level (*p* > 0.05 for all variables). However, the hemoglobin level (F = 3.45; *p* = 0.072), ANC (F = 3.01; *p* = 0.092), and WBC count (F = 2.81; *p* = 0.103) showed borderline trends toward significance, suggesting a possible influence of Brucella infection on these parameters.

Effect size estimates (partial eta-squared) further supported these observations. Hemoglobin had the greatest effect size (η^2^ = 0.095), followed by ANC (η^2^ = 0.084) and WBC count (η^2^ = 0.078), indicating small to moderate effects of Brucella infection on these hematological indices. In contrast, AMC (η^2^ = 0.034), PLT (η^2^ = 0.022), and ALC (η^2^ = 0.005) showed minimal effect sizes, reflecting the negligible influence of Brucella status on these parameters.

### 3.8. Diagnostic Performance of the Hematological Parameters

**Figure 2:**
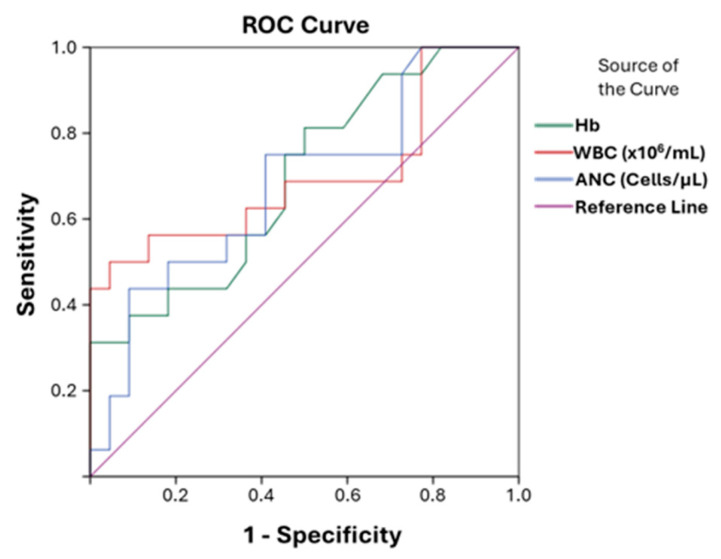
Receiver operating characteristic (ROC) curves for hematological parameters in predicting Brucella infection. The curves illustrate the diagnostic performance of hemoglobin (Hb), white blood cell count (WBC), and absolute neutrophil count (ANC) in distinguishing between culture-confirmed brucellosis and non-culture-confirmed brucellosis cases. The area under the curve (AUC) was 0.695 for Hb (95% CI: 0.53–0.86), 0.699 for WBC (95% CI: 0.52–0.88), and 0.68 for ANC (95% CI: 0.51–0.86), indicating fair discriminatory ability for all three parameters. The diagonal reference line represents an AUC of 0.5 (no discrimination).

Receiver operating characteristic (ROC) curve analysis was performed to evaluate the diagnostic performance of significantly different hematological parameters in distinguishing culture-confirmed brucellosis from non-culture-confirmed brucellosis cases (Figure 2, Table 6). The area under the ROC curve (AUROC) demonstrated fair discrimination for all three parameters. Hemoglobin had an AUROC of 0.695 (95% CI: 0.53–0.86), the WBC count had an AUROC of 0.699 (95% CI: 0.52–0.88), and the ANC had an AUROC of 0.680 (95% CI: 0.51–0.86), indicating that each parameter had a comparable and modest discriminatory ability to differentiate between culture-confirmed brucellosis and non-culture-confirmed brucellosis individuals.

The optimal cutoff value for hemoglobin concentration was <11.75 g/dL, yielding a sensitivity of 75% and a specificity of 54.5%. This cutoff demonstrated a positive predictive value (PPV) of 69.4% and a negative predictive value (NPV) of 61%, with an overall diagnostic accuracy of 68%, suggesting that hemoglobin is a moderately sensitive marker for Brucella detection.

For the WBC count, the optimal cutoff was <4.06 × 10^3^/µL, with high specificity (95.5%) but relatively low sensitivity (50%). This parameter exhibited an excellent PPV of 95.5%, indicating a strong confirmatory value when positive, although its NPV was modest (58%). The diagnostic accuracy was 69%, indicating that the WBC count is a useful rule-in test for Brucella infection.

An ANC cutoff of <1869.8 cells/µL demonstrated a sensitivity of 75% and a specificity of 59.1%, with a PPV and NPV of 71.6% and 63%, respectively. The overall diagnostic accuracy for ANC was 68%, indicating balanced sensitivity and specificity in detecting Brucella infection.

### 3.9. Predictive Model for Culture-Confirmed Brucellosis

Binary logistic regression analysis was performed to develop a prediction model for culture-confirmed brucellosis using hematological parameters as independent variables (Table 7). The analysis revealed that none of the hematological parameters emerged as independent, statistically significant predictors of culture-confirmed brucellosis in the multivariable model (*p* > 0.05 for all).

Hemoglobin demonstrated a negative regression coefficient (B = −0.26), indicating that lower hemoglobin levels were associated with higher odds of culture-confirmed brucellosis; however, this association was not statistically significant (*p* = 0.468). The adjusted odds ratio for hemoglobin was 0.77 (95% CI: 0.39–1.55), suggesting a nonsignificant trend.

Similarly, the WBC count was negatively correlated with culture-confirmed brucellosis (B = −0.47), with an adjusted odds ratio of 0.62 (95% CI: 0.29–1.33), but this difference was not statistically significant (*p* = 0.222). ANC, AMC, ALC, and PLT had regression coefficients near 0, with odds ratios near 1.0 and wide confidence intervals, indicating that these variables did not make a meaningful independent contribution to predicting Brucella infection after adjusting for other variables.

The constant term of the model was B = 4.24, corresponding to a baseline odds ratio of 69.73, although this was also not statistically significant (*p* = 0.197).

## 4. Discussion

The present single-center study provides a comprehensive evaluation of the demographic characteristics, clinical manifestations, microbiological profiles, treatment patterns, and hematological footprints of patients with pediatric brucellosis in an endemic region of Saudi Arabia. Our findings are largely consistent with previously published regional and international data and offer important insights into the diagnostic relevance of hematological parameters in routine pediatric practice.

The age distribution of the affected children in this study was relatively uniform, with a slight predominance among school-aged children. This observation is consistent with earlier reports demonstrating that children in this age group are more likely to be exposed to unpasteurized dairy products and infected livestock through household or environmental contact [1,2]. The observed male predominance also aligns with previous studies from Saudi Arabia and neighboring endemic regions, which found that boys tend to engage in greater outdoor activity and animal exposure, thereby increasing their risk of infection [4,11]. The fact that all patients were Saudi nationals reflects the endemic nature of brucellosis in the local population, as reported in national surveillance data [10,13].

The microbiological findings in this study demonstrated culture positivity in 42.1% of cases, comparable to previously reported yields for pediatric brucellosis. The relatively modest sensitivity of blood culture is well-recognized and may be influenced by intermittent bacteremia, prior antibiotic exposure, and technical limitations, particularly in children [15,16]. The considerable proportion of culture-negative or untested cases highlights the continued reliance on serological diagnosis in endemic settings, despite known limitations such as reduced sensitivity in early infection and variability in interpretation [14].

Consistent with earlier Saudi and regional studies, Brucella *melitensis* was the predominant species identified, reflecting its close association with sheep and goat reservoirs in the region [3,12]. The identification of mixed infections, although uncommon, underscores the complexity of exposure patterns in endemic areas and has been reported sporadically in pediatric cohorts [7].

The antimicrobial treatment patterns observed in this study were consistent with international and national recommendations. Rifampicin was the most commonly prescribed agent and was used in combination regimens to reduce relapse rates, in accordance with established pediatric treatment guidelines [22]. The use of cotrimoxazole, doxycycline, and aminoglycosides mirrors practices reported in other pediatric cohorts and supports appropriate antimicrobial stewardship in the study setting [4,17].

Clinically, fever was the most common presenting symptom, affecting nearly 90% of patients, consistent with previous studies that identified fever as the hallmark feature of pediatric brucellosis [1,6]. Musculoskeletal involvement, including bone and joint pain, was also highly prevalent, reflecting the known tropism of Brucella species for the reticuloendothelial and musculoskeletal systems [9]. Bleeding manifestations were uncommon but clinically significant, as they may indicate underlying hematological involvement and warrant careful evaluation.

One of the most important findings of this study was the significant reduction in hemoglobin levels among culture-confirmed brucellosis patients. These findings support existing evidence that anemia is the most common hematological abnormality in patients with pediatric brucellosis [5,7]. The underlying mechanisms are multifactorial and may include chronic inflammation, bone marrow suppression, and hypersplenism. Similarly, the observed reductions in WBC and ANC levels are consistent with previous reports describing leukopenia and relative neutropenia in infected children [8,9]. In contrast, monocyte, lymphocyte, and platelet counts did not differ significantly between groups, suggesting that brucellosis may selectively affect certain hematopoietic lineages.

The hematological abnormalities observed in pediatric brucellosis are likely multifactorial in origin. Brucella species are facultative intracellular pathogens that can invade and persist within cells of the reticuloendothelial system, including macrophages in the bone marrow, spleen, and liver [23,24]. Bone marrow involvement may result in the suppression of hematopoiesis, leading to reductions in one or more blood cell lineages [25,26,27]. In addition, chronic inflammatory responses induced by Brucella infection can alter iron metabolism and erythropoiesis through cytokine-mediated mechanisms, contributing to the development of anemia of inflammation [28,29]. Splenic enlargement, which is frequently observed in brucellosis, may further contribute to cytopenias through hypersplenism and increased sequestration of circulating blood cells [30,31]. Immune-mediated peripheral destruction of erythrocytes, leukocytes, and platelets has also been reported, likely resulting from the production of autoantibodies and immune complexes. In rare cases, hemophagocytic activity within the bone marrow and reticuloendothelial system has been described and may lead to severe pancytopenia. These mechanisms collectively explain the characteristic hematological manifestations of brucellosis and highlight the complex interaction between the pathogen and host immune response.

The absence of significant differences in hematological parameters across Brucella species further supports the hypothesis that host immune response and disease chronicity play a greater role in determining hematological changes than the infecting species does [1,2,32]. These findings are consistent with those of earlier studies demonstrating similar hematological patterns across different Brucella species.

Bone and joint pain was more frequently reported among patients with non-culture-confirmed brucellosis than among those with culture-confirmed brucellosis. These findings may reflect referral or selection bias, as children presenting with musculoskeletal symptoms are more likely to be evaluated for brucellosis even when alternative diagnoses are present. Similar diagnostic challenges have been described in endemic regions, where the nonspecific presentation of brucellosis overlaps with that of rheumatologic, infectious, and inflammatory conditions [3,4,33].

Multivariate ANOVA did not identify any single hematological parameter as independently significant at the 5% level; however, hemoglobin, WBC, and ANC showed borderline *p*-values and small-to-moderate effect sizes, collectively indicating a meaningful, although modest, hematological impact of Brucella infection. This finding is in line with prior studies that describe hematologic abnormalities as frequent but not individually diagnostic [7,9,34]. ROC curve analysis further demonstrated fair discriminatory performance for hemoglobin, WBC, and ANC, with AUROC values ranging from approximately 0.68–0.70. Hemoglobin and ANC cutoffs provided reasonable sensitivity, whereas a low WBC cutoff showed excellent specificity and positive predictive value, suggesting that markedly reduced WBC counts may be particularly useful as a rule-in feature when combined with epidemiologic and clinical suspicion. Nonetheless, these indices alone were insufficient for reliable diagnosis, underscoring that hematological changes should be viewed as supportive rather than standalone diagnostic criteria and interpreted alongside serological and microbiological data [14,15].

Logistic regression analysis confirmed that none of the hematological parameters independently predicted culture-confirmed brucellosis in the multivariable model. This finding reinforces the understanding that hematological changes in brucellosis are multifactorial and influenced by disease stage, host immunity, nutritional status, and coexisting conditions [16,35]. The wide confidence intervals observed also reflect the limited sample size and highlight the need for larger prospective studies to validate these findings. Taken together, the data suggest that while anemia and neutropenia represent characteristic hematological footprints of pediatric brucellosis, they function best as components of a broader diagnostic framework rather than as isolated predictors.

### 4.1. Study Limitations

This study has several limitations that should be acknowledged. Its retrospective, single-center design and relatively small sample size may limit generalizability and reduce the statistical power to detect subtle associations. Incomplete culture performance in a subset of patients, potential prior antibiotic exposure, and reliance on serological criteria may also have led to the misclassification or underestimation of true infection status. Furthermore, the absence of longitudinal hematological follow-up in all patients precluded a detailed assessment of the temporal evolution and resolution of the cytopenias. The relatively small number of culture-confirmed cases may have limited the robustness of the multivariable analyses and affected the stability of the ROC-derived cutoff values. Therefore, the MANOVA, logistic regression, and ROC analyses should be considered exploratory and hypothesis-generating rather than definitive. Larger prospective multicenter studies are required to validate the observed associations, confirm the diagnostic performance of the identified hematological parameters, and determine whether combinations of hematological markers provide greater diagnostic accuracy than individual parameters. Despite these limitations, this study provides valuable regional data on the hematological profile of pediatric brucellosis and highlights practical diagnostic implications for clinicians in endemic areas.

### 4.2. Clinical Implications

Despite these limitations, this study has significant clinical implications. The finding that hemoglobin levels, WBC counts, and ANCs are notably altered in patients with pediatric brucellosis indicates that routine complete blood counts can help raise clinical suspicion in suitable epidemiological contexts. The high specificity of leukopenia (<4.06 × 10^3^/µL) makes it a useful rule-in test, especially in primary care settings where serological testing might be delayed. Clinicians in endemic areas should stay alert for brucellosis in children with unexplained fever, cytopenias, or musculoskeletal symptoms, and should not rely solely on symptom patterns to rule out the diagnosis.

## 5. Conclusions

This study demonstrated that pediatric brucellosis remains an important health concern in the Hail region of Saudi Arabia, with Brucella melitensis being the predominant species. This disease is frequently associated with significant hematological abnormalities, including anemia, leukopenia, and neutropenia, which can serve as useful supportive diagnostic indicators in endemic settings. Although no single hematological parameter independently predicts infection, the concurrent presence of low hemoglobin levels, low WBC counts, and low ANC may support clinical suspicion and prompt appropriate serological testing. These findings support integrating routine hematological evaluation into the diagnostic workup of suspected pediatric brucellosis and highlight the need for continued public health efforts to reduce disease burden in endemic regions.

## Figures and Tables

**Table 1:** Distribution of cases according to baseline characteristics.

Baseline Characteristics	No.	%
Age		
3–6 years	10	26.3%
>6–9 years	11	28.9%
>9–12 years	10	26.3%
>12–15 years	7	18.4%
Sex		
Male	22	57.9%
Female	16	42.1%

**Table 2:** Comparison of hematological parameters between the non-culture-confirmed and culture-confirmed brucellosis patients.

Hematological Parameter	Non-Culture-Confirmed Brucellosis (*n* = 22)		Culture-Confirmed Brucellosis (*n* = 16)		Unpaired *t*-Test	
	Mean	SD	Mean	SD	t-Value	*p*-Value
Hemoglobin	12.1	1.4	10.8	1.7	−2.43	0.020 *
WBC (×10^3^/µL)	6.2	1.7	5.0	2.0	2.06	0.046 *
ANC (cells/µL)	2025.9	789.2	1475.8	745.0	2.17	0.037 *
AMC (cells/µL)	509.6	209.2	406.3	271.5	−1.29	0.206
ALC (cells/mm^3^)	3215.6	1369.3	3544.0	3179.5	0.42	0.681
PLT (×10^3^/µL)	248.1	109.1	208.9	101.8	−1.12	0.268

* *p*-value is significant at <0.05. WBC—white blood cell; ANC—absolute neutrophil count; AMC—absolute monocyte count; ALC—absolute lymphocyte count; PLT—platelets.

**Table 3:** Comparison of hematological parameters among *Brucella* types.

Hematological Parameter	*B. melitensis* (*n* = 11)		*B. abortus* (*n* = 2)		*B. melitensis* and *B. abortus* (*n* = 3)		ANOVA	
	Mean	SD	Mean	SD	Mean	SD	F-Value	*p*-Value
Hemoglobin	10.9	1.7	11.8	0.6	9.4	2.3	1.05	0.377
WBC (×10^3^/µL)	5.2	2.0	3.5	0.5	5.3	3.2	0.62	0.556
ANC (cells/µL)	1575.3	817.0	1250.0	622.3	1105.0	318.2	0.41	0.671
AMC (cells/µL)	414.2	269.1	290.0	141.4	475.0	502.0	0.23	0.800
ALC (cells/mm^3^)	3193.6	1396.6	1820.0	905.1	7195.0	9340.9	1.89	0.193
PLT (×10^3^/µL)	204.5	93.1	305.5	71.4	138.5	161.9	1.48	0.264

WBC—white blood cell; ANC—absolute neutrophil count; AMC—absolute monocyte count; ALC—absolute lymphocyte count; PLT—platelets; ANOVA—analysis of variance.

**Table 4:** Association between clinical symptoms and culture-confirmed brucellosis.

Symptoms	Non-Culture-Confirmed Brucellosis (*n* = 22)		Culture-Confirmed Brucellosis (*n* = 16)		Significance		OR	95% CI
	Mean	SD	Mean	SD	Chi sq.	*p*-Value		
Fever								
Present	19	55.9%	15	44.1%	0.54	0.464	0.42	(0.04–4.48)
Not Present	3	75.0%	1	25.0%				
Fatigue								
Present	11	64.7%	6	35.3%	0.59	0.444	1.67	(0.45–6.19)
Not Present	11	52.4%	10	47.6%				
Bone/joint pain								
Present	20	74.1%	7	25.9%	10.02	0.002 *	12.86	(2.22–74.54)
Not Present	2	18.2%	9	81.8%				
Sweating								
Present	9	75.0%	3	25.0%	2.11	0.147	3.00	(0.66–13.66)
Not Present	13	50.0%	13	50.0%				
Bleeding								
Present	1	20.0%	4	80.0%	3.39	0.066	0.14	(0.01–1.43)
Not Present	21	63.6%	12	36.4%				

* *p*-value is significant at <0.05. OR—odds ratio; CI—confidence interval.

**Table 5:** Multivariate analysis of hematological parameters in culture-confirmed and non-culture-confirmed brucellosis.

Source: Culture Confirmation Status			
Hematological Parameter	F-Value	*p*-Value	Partial η^2^
Hemoglobin	3.45	0.072	0.095
WBC (×10^3^/µL)	2.81	0.103	0.078
ANC (cells/µL)	3.01	0.092	0.084
AMC (cells/µL)	1.15	0.291	0.034
ALC (cells/mm^3^)	0.17	0.681	0.005
PLT (×10^3^/µL)	0.74	0.396	0.022

WBC—white blood cell; ANC—absolute neutrophil count; AMC—absolute monocyte count; ALC—absolute lymphocyte count; PLT—platelets, Partial η^2^—partial eta-squared effect size.

**Table 6:** ROC analysis of hematological parameters for predicting culture-confirmed brucellosis.

Parameter	Hb Level		WBC Level		ANC Level	
	Value	95% CI	Value	95% CI	Value	95% CI
AUROC	0.695	(0.53–0.86)	0.699	(0.52–0.88)	0.68	(0.51–0.86)
Optimum cut off	Hb < 11.75 g/dL		WBC < 4.06 × 10^3^/µL		ANC < 1869.8 cells/µL	
Sensitivity	75	(61.2–88.8)	50	(34.1–65.9)	75	(61.2–88.8)
Specificity	54.5	(38.7–70.3)	95.5	(88.9–100.0)	59.1	(43.5–74.7)
PPV	69.4	(54.7–84.1)	95.5	(88.9–100.0)	71.6	(57.3–85.9)
NPV	61	(45.5–76.5)	58	(42.3–73.7)	63	(47.6–78.4)
DA	68	(53.2–82.8)	69	(54.3–83.7)	68	(53.2–82.8)

AUROC—area under the ROC curve; PPV—positive predictive value; NPV—negative predictive value; DA—diagnostic accuracy.

**Table 7:** Logistic regression analysis of hematological parameters associated with culture-confirmed brucellosis.

Dependent: Culture-Confirmed Brucellosis					
Hematological Parameter	B	SE	*p*-Value	Exp(B)	95% CI for Exp(B)
Hb	−0.26	0.35	0.468	0.77	0.39–1.55
WBC	−0.47	0.39	0.222	0.62	0.29–1.33
ANC	0.00	0.00	0.960	1.00	1.00–1.00
AMC	0.00	0.00	0.814	1.00	1.00–1.00
ALC	0.00	0.00	0.256	1.00	1.00–1.00
PLT	0.00	0.00	0.927	1.00	0.99–1.01
Constant	4.24	3.29	0.197	69.73	

B—logistic regression coefficient; SE—standard error; Exp(B)—exponentiation of B (odds ratio, OR); CI—confidence interval.

## Data Availability

The original contributions presented in this study are included in the article. Further inquiries can be directed to the corresponding author.

## Abbreviations

The following abbreviations are used in this manuscript:

ALC	Absolute lymphocyte count
AMC	Absolute monocyte count
ANC	Absolute neutrophil count
ANOVA	Analysis of variance
AUROC	Area under the ROC curve
B	Logistic regression coefficient
CI	Confidence interval
DA	Diagnostic accuracy
MANOVA	Multivariate analysis of variance
NPV	Negative predictive value
PPV	Positive predictive value
ROC	Receiver operating characteristic
SE	Standard error
STAT	Standard tube agglutination test
WBC	White blood cell
